# Oral and Craniofacial Manifestations and Two Novel Missense Mutations of the NTRK1 Gene Identified in the Patient with Congenital Insensitivity to Pain with Anhidrosis

**DOI:** 10.1371/journal.pone.0066863

**Published:** 2013-06-14

**Authors:** Li Gao, Hao Guo, Nan Ye, Yudi Bai, Xin Liu, Ping Yu, Yang Xue, Shufang Ma, Kewen Wei, Yan Jin, Lingying Wen, Kun Xuan

**Affiliations:** 1 Department of Pediatric Dentistry, School of Stomatology, Fourth Military Medical University, Xi'an, Shaanxi Province, People's Republic of China; 2 Department of Dentistry, Hospital of PLA 309, Beijing, People's Republic of China; 3 Department of Orthodontics, School of Stomatology, Fourth Military Medical University, Xi'an, Shaanxi Province, People's Republic of China; 4 Institute of Genomic Medicine, Wenzhou Medical College, Wenzhou, Zhejiang Province, People's Republic of China; 5 Department of Oral Biology, School of Stomatology, Fourth Military Medical University, Xi'an, Shaanxi Province, People's Republic of China; 6 Department of Endodontics, School of Stomatology, Fourth Military Medical University, Xi'an, Shaanxi Province, People's Republic of China; 7 Department of Oral Histology and Pathology, School of Stomatology, Fourth Military Medical University, Xi'an, Shaanxi Province, People's Republic of China; CNRS UMR7275, France

## Abstract

Congenital insensitivity to pain with anhidrosis (CIPA) is a rare inherited disorder of the peripheral nervous system resulting from mutations in neurotrophic tyrosine kinase receptor 1 gene (*NTRK1*), which encodes the high-affinity nerve growth factor receptor TRKA. Here, we investigated the oral and craniofacial manifestations of a Chinese patient affected by autosomal-recessive CIPA and identified compound heterozygosity in the *NTRK1* gene. The affected boy has multisystemic disorder with lack of reaction to pain stimuli accompanied by self-mutilation behavior, the inability to sweat leading to defective thermoregulation, and mental retardation. Oral and craniofacial manifestations included a large number of missing teeth, nasal malformation, submucous cleft palate, severe soft tissue injuries, dental caries and malocclusion. Histopathological evaluation of the skin sample revealed severe peripheral nerve fiber loss as well as mild loss and absent innervation of sweat glands. Ultrastructural and morphometric studies of a shed tooth revealed dental abnormalities, including hypomineralization, dentin hypoplasia, cementogenesis defects and a dysplastic periodontal ligament. Genetic analysis revealed a compound heterozygosity- c.1561T>C and c.2057G>A in the NTRK1 gene. This report extends the spectrum of *NTRK1* mutations observed in patients diagnosed with CIPA and provides additional insight for clinical and molecular diagnosis.

## Introduction

Congenital insensitivity to pain with anhidrosis (CIPA, MIM256800) is also known as hereditary sensory and autonomic neuropathy type IV (HSAN-IV). CIPA is a rare autosomal recessive disorder first described nearly 50 years ago [1], [2]. Thus far, several hundred cases of CIPA have been reported, and most of them are in pediatric patients [3]. Primary clinical characteristics of CIPA include congenital insensitivity to pain, particularly in extremities and oral tissue, self-mutilating behavior, temperature-sensing defects, and mental retardation [4]. CIPA-affected children fail to sweat, have unexplained episodes characterized by recurrent traumatic and thermal injuries, and are typically below-average height and weight for their age. Their self-mutilating behavior primarily involves the orofacial region. Oral manifestations include premature tooth loss, lacerations and ulcerations of oral soft tissues, limited ability to open the mouth due to intra-oral scarring, severe dental attrition and tooth luxation, and a high incidence of dental caries [4], [5]. To our knowledge, dental histopathological manifestations have never been reported, and it is still unclear whether oral and dental phenotypes are associated with corresponding histopathological abnormalities.

The diagnosis of CIPA is usually based on clinical examination and neurological evaluations. Neuropathological studies have revealed a severe loss of unmyelinated and small-diameter nerve fibers of the afferent neurons, defective development of small nociceptive neurons in the dorsal root ganglia (DRG), and the abnormal innervation of eccrine sweat glands by cholinergic sympathetic fibers [6], [7]. Some studies have demonstrated an association between mutations in the neurotrophic tyrosine kinase receptor type 1 (*NTRK1*) and CIPA. The *NTRK1* gene encodes the high-affinity receptor for NGF regulating the neural development and differentiation [8]. To date, more than 50 *NTRK1* mutations have been reported in different ethnic groups [9], [10]. The present paper reports the oral and craniofacial manifestations of a Chinese child with CIPA and reveals two new missense mutations within the *NTRK1* gene.

## Results

### Clinical finding

The patient was born after a normal pregnancy and delivery. From the third month of life onward, he experienced recurrent unexplained fevers. An absence of sweating with dry warm skin was consistently noted during the febrile periods. Meanwhile, he was also diagnosed with congenital corneitis and submucous cleft palate. The patient began biting his tongue and fingers after his teeth erupted at the age of 8 months. His parents and physicians began to suspect a developmental abnormality when he failed to cry during immunization or other painful stimuli although he could feel pressure and touch. His height and weight were below normal for his age. Other members of the patient's family did not show similar clinical symptoms.

**Figure 1:**
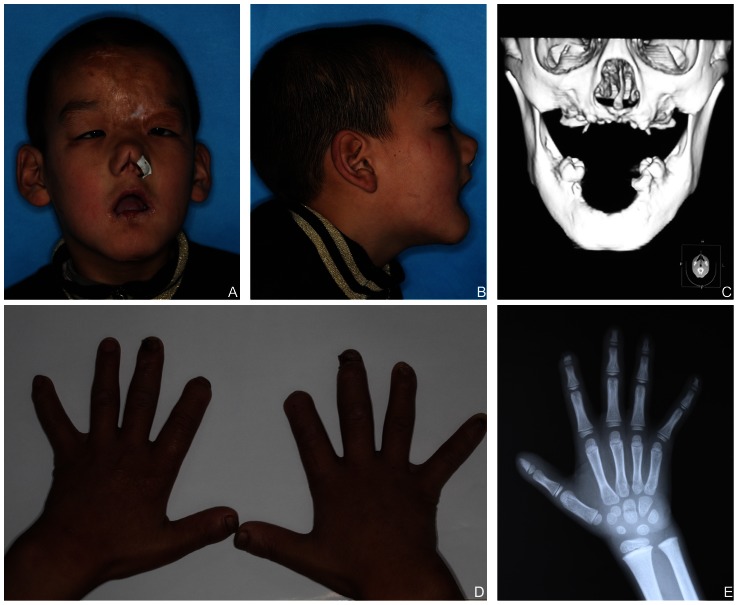
Clinical features of the proband. (A) Scratch marks, erosions, cicatrization healing (scars) due to self-inflicted soft tissue trauma, nasal columella defect and keratitis. (B) Abnormal profile, limitation of mouth opening. (C) A cranial CT scan and reconstruction of head. (D) Lichenification, missing tips of right index fingers, and the deformed nail and left digitus minimus. (E) Radiograph of the hand and wrist bones.

Clinical examination revealed that the proband presented a developmental delay. Scratch marks, erosions and cicatrization healing (scars) due to self-inflicted soft tissue trauma were seen on his face. He had congenital nasal defects, including depressed nasal bridge, malformed nasal apex, narrow nostril, dysmorphic columella and deviated nasal septum. And he had skin erosions on the left nasal alae and corneal ulcerations in the right eye as a result of scratching (Figure 1-A, B). A cranial CT scan showed a submucous cleft palate and alveolar bone loss of the maxilla and mandible (Figure 1-C). His hands and fingers also showed signs of biting. The tips of his right index fingers were missing, he was exhibiting amputations of the fingers caused by osteomyelitis in the distal region, and the left digitus minimus extended outward due to old fractures (Figure 1-D). Radiographs of the hand and wrist bones suggested that the bone age measurement was normal for his age and proved his bone fracture histories (Figure 1-E). Xerosis, thick and calloused skin, lichenification of the palms, dystrophic nails, and areas of hypotrichosis on the scalp were present in the patient. The proband presented with waddling gait- exaggerated alternation of lateral trunk movements with an exaggerated elevation of the hip, caused by valgus knee and hypotonia.

Sensory examination revealed that pain and temperature sensing were absent, while deep tendon reflexes and his sense of touch were primarily intact. Electrophysiological studies suggested a peripheral neuropathy that was predominantly axonal and sensory and that showed greater involvement of the lower than the upper limbs. Electromyography and motor and sensory nerve conduction velocities were within normal limits. An intradermal injection of 0.05 ml 1∶1000 histamine solution resulted in the expected weal without any axon flare. No sweat was formed upon sweat induction with pilocarpine. Psychomotor development tests performed on Vienna Test System (VTS), showed mental retardation (Intelligence Test Score = 70, normal range for 7-year-old Chinese children 80∼100) and mild delays in his motor milestones (Motor Performance Series Score  = 77, normal range for 7-year-old Chinese children 95∼115). Laboratory examinations could not detect abnormalities. Chromosome karyotype analysis was normal.

**Figure 2:**
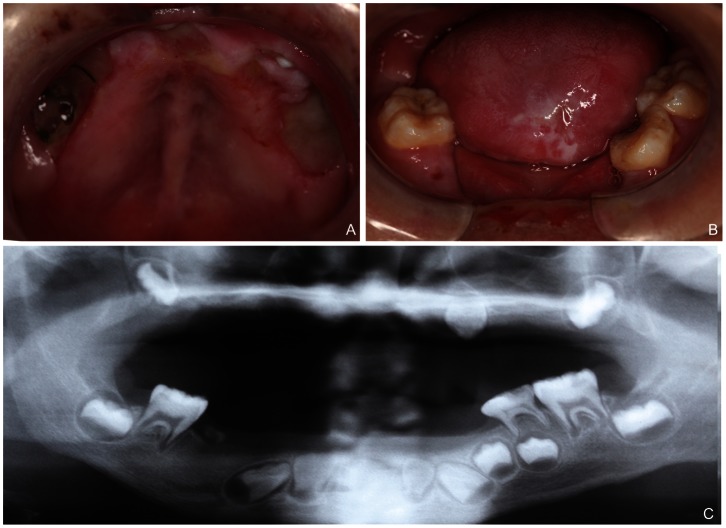
Oral manifestations of the proband. (A, B) Early teeth loss, the laceration and ulceration of oral soft tissues, dental attrition and dental caries. (C) Panoramic radiographs showing alveolar bone loss of the maxilla and mandible, exfoliation of 15 permanent teeth, and short root anomaly of the existing teeth.

Oral examination revealed erosions, scars and hyperkeratosis of soft tissues, including the tongue, lips, gingiva, and palatal and buccal mucosa (Figure 2-A). The patient had a decreased ability to open his mouth that was attributed to a fibrous band of scar tissue in the cheeks and lips. Early tooth loss was noted, including almost all primary teeth except for a left mandibular primary second molar that was carious and mobile and some permanent teeth (Figure 2-A,B). Panoramic radiographs showed that 15 permanent teeth were missing, including lower central incisors and right premolars, all maxillary teeth except for second molars and the left canine. What was more, the existed teeth exhibited short root anomalies (Figure 2-C). The proband's guardians testified that those missing teeth were not congenitally lost but had erupted and were then lost by autoextraction or self-mutilation.

### Histopathological examination

**Figure 3:**
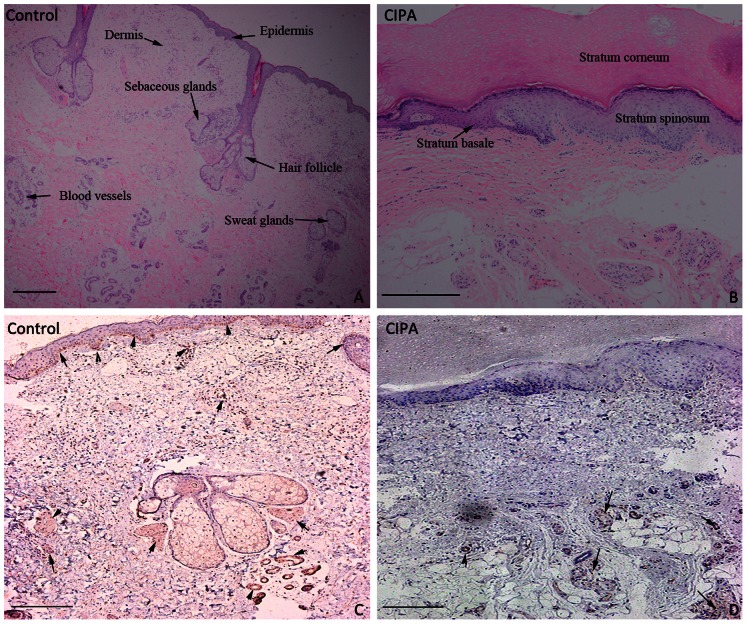
Histopathological examination of a skin biopsy. (A) Normal epidermis and appendages in healthy control samples (H&E staining). (B) Hyperkeratosis, acanthosis and irregular stratum basale, few sweat glands, hair follicles and sebaceous glands in the CIPA patient's samples (H&E staining). (C, D) NSE immunoreactivity stains of control and CIPA patient's sections. Arrow indicates the area with positive staining. (Immunohistochemical staining, Bar = 100 µm)

A skin biopsy revealed an aberrant hyperplastic epidermis, including hyperkeratosis, acanthosis and an irregular stratum basale. H&E stains showed a reduction in the number of sweat glands, hair follicles, blood vessels and sebaceous glands (Figure 3-A, B). NSE immunoreactivities are usually associated with both sensory and autonomic nerves of the skin, such as free nerve endings in the epidermis and dermis, sensory corpuscles, Merkel cells and sweat glands [11]. NSE immunohistochemistry showed that the density of NSE immunoreactivity stains was significantly decreased in the patient's skin when compared to healthy skin (Figure 3-C, D).

**Figure 4:**
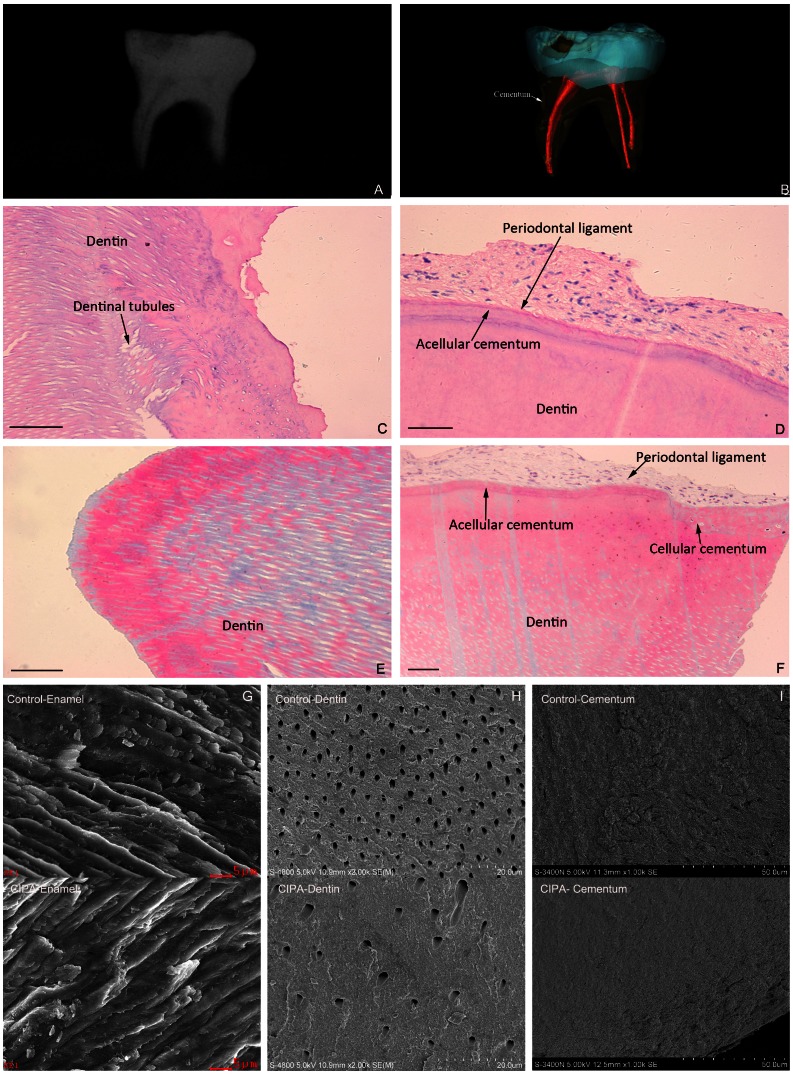
Pathological examination of the patient's exfoliated primary tooth. (A) The dental x-rays showing a normal crown, thin roots and smaller pulp chamber. (B) Micro CT scanning and reconstruction of the tooth showing the indistinct cementodentinal junction and the irregular cementum. (C, D) H&E stained tooth tissue showing that some dentinal tubules were in a disorganized state near the dentino-cemental junction. In addition, the acellular cementum was thin and the cellular cementum was partially absent, and the periodontal ligament was loosely organization (Bar = 100 µm). (E, F) Masson's stained tooth tissue showing dentinal tubules with significantly greater diameters, dentine in a state of hypomineralization and hypomaturation, a hypoplastic cementum and seemingly sparse periodontal ligament fibers (Bar = 100 µm). (G) The CIPA patient's enamel prisms showing the disorientation and confusion in hydroxyapatite crystal growth (SEM, Bar = 5 µm). (H) The significantly abnormal distribution in density and diameter of the dentinal tubules compared with the control samples (SEM, Bar = 20 µm). (I) The root surface of the patient's teeth showing discontinuous mineralization, a thinner cementum and fewer attached fiber occupancies. (SEM, Bar = 50 µm).

Radiographs of the exfoliated tooth sample revealed a normally sized and shaped crown except for a carious cavity, two thin roots and a smaller pulp chamber (Figure 4-A), and it was difficult to distinguish from the dentin and cementum by Micro CT (Figure 4-B). H&E-stained tooth tissue showed that some dentinal tubules were in a disorganized state near the dentino-cemental junction, that the acellular cementum was thin, the cellular cementum was partially absent, and the periodontal ligament displayed a loose, disordered organization (Figure 4-C, D). Masson's stained tooth tissue revealed dentinal tubules with significantly greater diameters, dentine in a state of hypomineralization and hypomaturation, a hypoplastic cementum and seemingly sparse periodontal ligament fibers (Figure 4-E, F).

### SEM- EDS examination

Under the scanning electron microscope, the patient's enamel prisms showed slight disorientation and confusion in hydroxyapatite crystal growth (Figure 4-G). The distribution density of the dentinal tubules was significantly abnormal when compared with control subjects with sparse dentinal tubules that had wider diameters, and expanded peritubular dentin spaces (Figure 4-H). The shape of the enamelo-dentinal junction (EDJ) in CIPA patients is generally wider and straighter. Hypoplastic root cementum in this CIPA patient featured discontinuous mineralization, a thinner cementum and less attached fiber occupancies (Figure 4-I).

**Figure 5:**
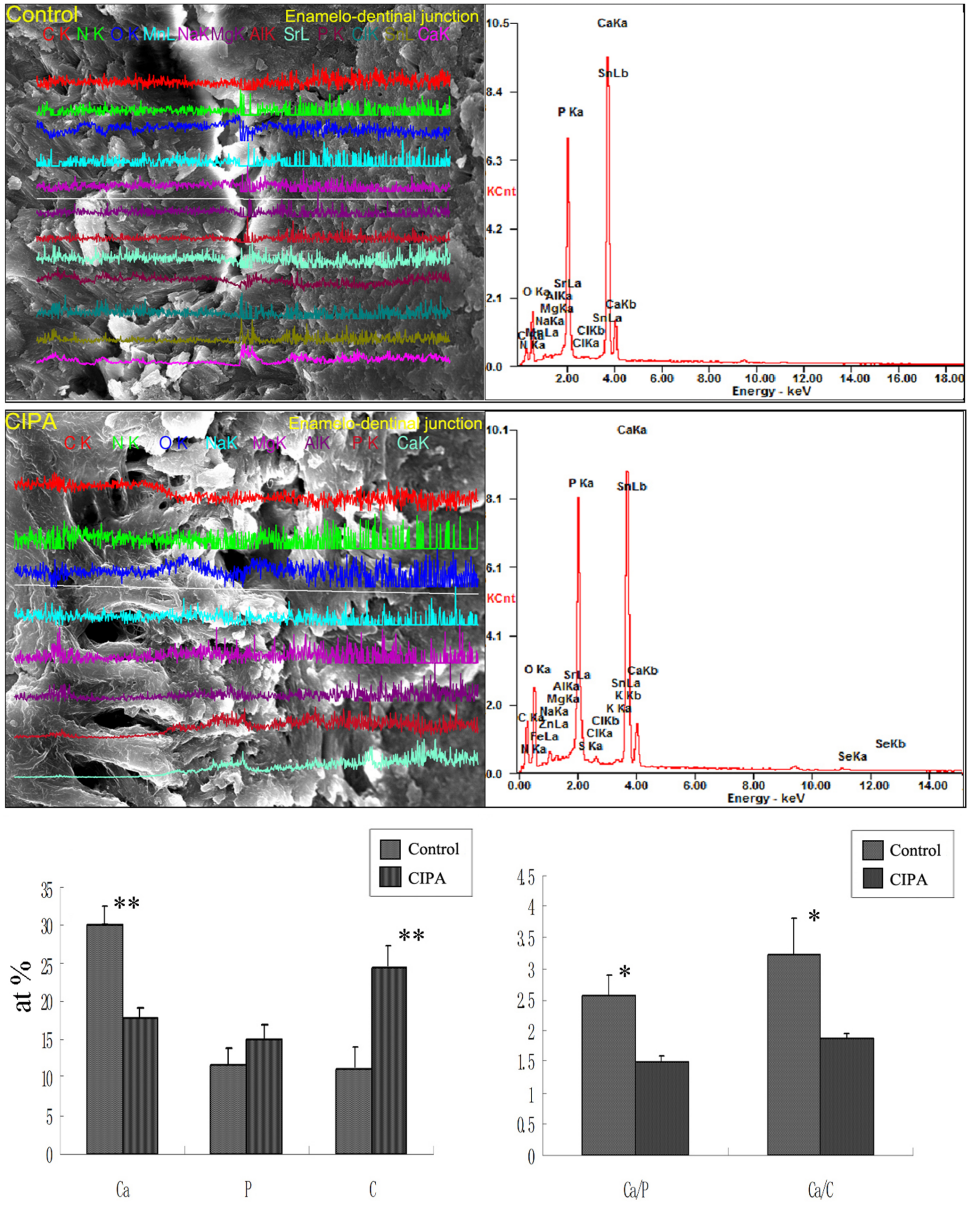
Spot elemental analyses for ultrastructural and quantitative changes in the patient's tooth. Compared to healthy controls, significant differences were detected in the level of Ca and the Ca/P and Ca/C ratios in the EDJ of the patient (*p*<0.05).

Ultrastructural and quantitative changes of the enamel and dentin were evaluated by micrograph, and viewed under x-ray energy dispersive analysis. Lines vertical to the EDJ at increasing incremental distances of 64 µm were scanned, and the collected spectra were used to draw atomic ratio plots. Compared to healthy controls, significant differences were detected in the level of Ca, P, Carbon (C) and Ca/P, Ca/C ratios in the EDJ of the patient (Figure 5). There were similar atom ratios of nitrogen, magnesium, sulfur, chlorine, manganese, and strontium between the CIPA and normal tooth.

### Mutation analysis

**Figure 6:**
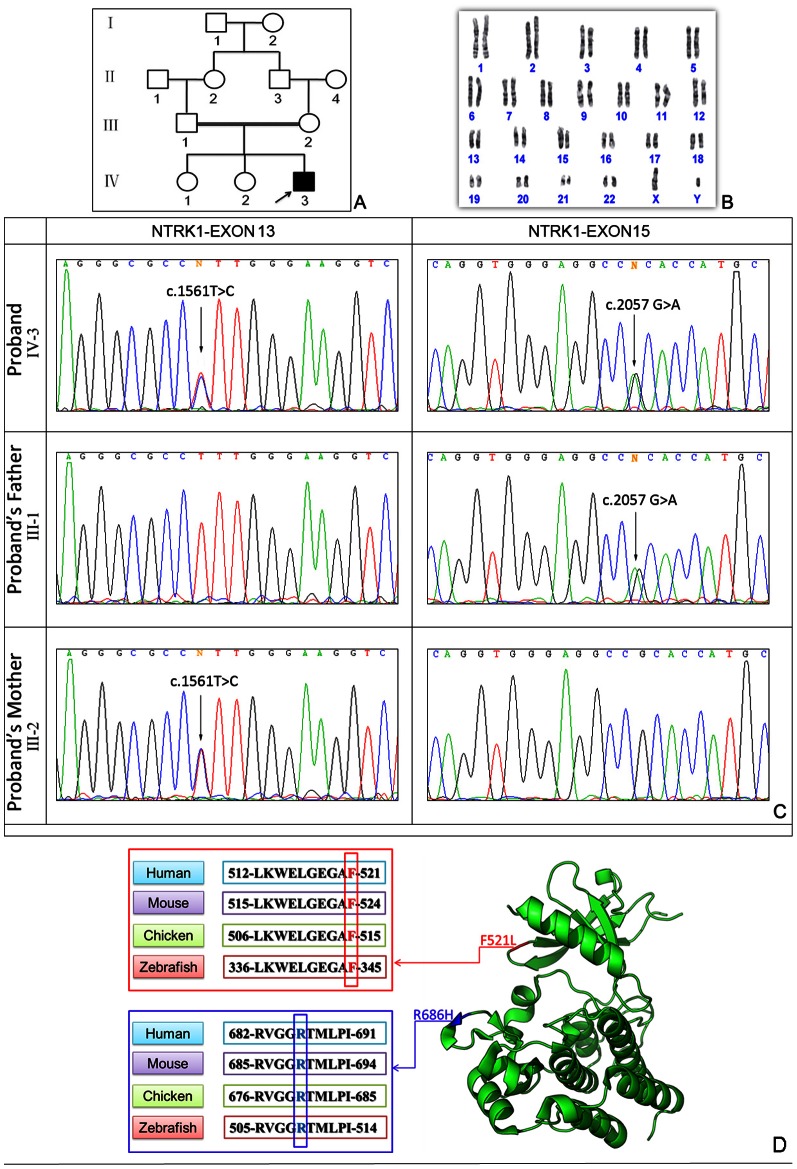
Genetic analysis of the CIPA family: (A) pedigree of the family. (B) Chromosome karyotype analysis was normal. (C) Sequencing chromatographs of genomic DNA from the proband and his parent are shown and reveal double heterozygous missense mutations- c.1561T>C in exon 13 and c.2057 G>A in exon 15 of the *NTRK1* gene in the proband. Mutation-c.1561T>C was found in the proband's father, and mutation- c.2057 G>A was found in his mother. (D) Multiple sequence alignment: F521 and R686 of human *NTRK1*, indicated by the red frame and blue frame respectively, are evolutionarily conserved as shown in four representative species. The crystal structure of amino acids 497–795 of the human NTRK1, located in the catalytic domain, is shown in green ribbons. Two mutated p.F521L (in red) and p.R686H (in blue) are all labeled.

The human *NTRK1* gene on chromosome 1q21–q22 (GenBank acc. no., NM_002529. 3) encodes a receptor tyrosine kinase for nerve growth factor and is responsible for an autosomal recessive genetic disorder. Sequencing analysis of the *NTRK1* gene in the proband revealed a compound heterozygote mutations, namely c. 1561T>C in exon 13 and c.2057 G>A in exon 15 inherited from each of his parents, which predicts amino acid substitutions p.F521L (phenylalanine acid→leucine) and p.R686H (arginine→histidine) (Figure 6). Multiple sequence alignment showed that F521 and R686 in the NTRK1 are evolutionarily conserved from zebrafish to humans (Figure 6-D). The three-dimensional structural model was presented using the PyMOL Molecular Graphics System, which showed that F521L (c.1561T>C) was located in the G-loop (amino acids 517–522), and R686H (c.2057 G>A) was shown in the middle of the activation segment (amino acids 671–696). To exclude the possibility of a rare single nucleotide polymorphism (SNP) in the Han population, 100 unrelated normal individuals were sequenced and neither mutation was found. After searching the SNP database and the human inherited peripheral neuropathies mutation database (http://www.molgen.ua.ac.be/CMTMutations/), we found that c.1561T>C (p.F521L) and c.2057 G>A (p.R686H) are absent from the two databases, indicating that the patient carries two novel missense mutations.

## Discussion

In this study we described the oral and dental manifestations of a Chinese child with CIPA and identified two missense mutations in the *NTRK1* gene. The patient has typical features, including insensitivity to pain, anhidrosis and mental retardation, confirming the diagnosis of CIPA. Histopathological examinations showed the absence of epidermal innervation, loss of most dermal innervation and hypoplastic dermal sweat glands without innervation. In addition, genetic examinations showed that the proband was in a state of compound heterozygosity, having two different mutations in the *NTRK1* gene on separate chromosomes. Although some degree of phenotypic variability was observed in the CIPA patients with proven *NTRK1* mutations, overall the clinical presentation corresponds to a readily recognizable syndrome that is genetically homogenous [12], [13]. In this case, the patient's parents and sisters were not affected as the carriers, indicating that the segregation of the mutations is consistent with a recessive disease. But this CIPA patient also exhibited some craniofacial manifestations which had never been documented in previous publications, such as nasal malformation, submucous cleft palate and developmental abnormalities of teeth. It is still uncertain whether the patient's disease was caused by single gene (*NTRK1*) disorder or multifactorial and polygenic (complex) disorders.

NTRK1 protein (TRKA) is a tyrosine kinase receptor comprising an extracellular domain involved in NGF binding, a single transmembrane region, a juxtamembrane domain, a tyrosine kinase domain (TKD), which is important for signal transduction, and a C-terminal tail [14]. Some studies identified TRKA tyrosine residues-470, 670, 674, 675 and 785 as autophosphorylation sites critical for activating intracellular signaling [15], [16]. The naturally occurring NTRK1 missense mutations close to crucial tyrosine residues G571R, R643W, R648C, D674Y, G708S and R774P, were detected in some CIPA patients, demonstrating that these conserved residues are important for TRKA activity [9], [17], [18]. In our studies, the 3D structural model analysis showed that F521L was located in the G-loop (also termed ATP phosphate-binding P loop), which could controls nucleotide affinity/specificity and g-phosphoryl transfer rate. R686H was located in the activation segment (amino acids 671–696), which is a main regulatory domain. A mutation in this domain inactivates the kinase activity by blocking the ATP accessibility and preventing the protein substrate to bind correctly [17], [19]. Our patient's phenotype suggests that the combination of two mutated residues could lead to a dramatic change in the biological function of TRKA.

The most important characteristic of CIPA is the self-mutilating behavior that leads to facial and oral soft tissues lesions and premature tooth loss. Our patient exhibited some developmental defects, including congenital corneitis, submucous cleft palate, nasal defect and abnormal teeth, suggesting that the NTRK1 mutations may affect not only innervation but the development of oro-facial tissues as well. NGF/TRKA can promote the proliferation of bone marrow mesenchymal stem cells [20]. It also plays a role in the regulation of preskeletal differentiation during craniofacial development in mice [21]. Additionally, NGF/TRKA can promote cell proliferation and the cell cycle progression of corneal epithelial cells by activating cyclin D via the PI3K/Akt and MAPK/Erk signaling pathways [22]. The NGF/TRKA pathway controls cranial neural crest cell fate specification and eventual differentiation into dental organs [23]. So *NTRK1* might be extensively involved in multi-systemic development and could be associated with tissue patterning. Although previous studies showed NGF/TRKA pathway could play an important role in craniofacial development, it still needs further functional studies to explore the correlation with *NTRK1* mutations and craniofacial defects. Moreover, the patient's parents were consanguineous and some of this patient's craniofacial features, nasal malformation and cleft palate, were not presented in other published individuals with CIPA. Therefore, further studies of array comparative genomic hybridization (array CGH) should be performed to exclude recessive disorders and dysmorphisms caused by other genomic imbalance.

To our knowledge, dental ultrastructure and micromorphology of CIPA patients have not been examined in previous reports. We first detected some dental abnormalities, including hypomineralization, thin teeth roots, dentin hypoplasia, cementogenesis defects and dysplastic periodontal ligament. Dental hypomineralization might increase the susceptibility to dental caries. We speculated further that dentin hypoplasia and hypomineralization could also contribute to dental attrition. Some studies showed that, as a potent promoter of mineralization during dentin formation, NGF significantly stimulates DNA synthesis and mineral deposition in cultures of human pulp cells, and induces differentiation of odontoblast-lineage cells with subsequent biomineralization in vitro[24], [25], [26]. The features of shed teeth in CIPA patient suggest that NTRK1 could play an important role in root formation. Some studies have shown that TRKA is a convenient marker for identifying and characterizing Hertwig's root sheath and epithelial rests of Malasseze of the forming root [27], [28]. Moreover, NGF/TRKA expressed in human periodontal tissue may contribute to the regeneration and the innervation of periodontal tissue [29], [30]. These results highlight the importance of the NGF/TRKA pathway during tooth root development. The common oral sign of CIPA disease, premature loss of teeth, may not only have attributed to self-mutilating behavior but also to the developmental defects caused by NTRK1 mutation.

In conclusion, we identified two novel heterozygous missense mutations in a Chinese patient with CIPA. Systemic disorders and oral manifestations may be associated with these mutations in the NTRK1 gene. Further studies should be performed to explore the effects of these mutations on oral-craniofacial development.

## Materials and Methods

### Participants and Clinical Data

This study was reviewed and approved by the Institutional Review Board and the Ethics Committee of the Fourth Military Medical University and was conducted after written consent was obtained from the participant's parents. Written informed consent was obtained from the parents of the patient (as outlined in PLOS consent form) to publish the case details. The patient, the son of a consanguineous marriage between first cousins, was a 6-year-old boy who exhibited pain insensitivity and loss of multiple primary and permanent teeth. All individuals of the patient's family underwent evaluation of their full medical history evaluation and comprehensive physical examinations. The patient was thoroughly examined, including comprehensive neurophysiological assessment and oral examination. A pedigree of his family was created through clinical examinations and interviews with all available family members. The diagnosis was further verified by laboratory assays and genetic tests. Furthermore, 100 unrelated healthy individuals (including different ages, gender equality) were taken as control subjects to exclude the possibility of frequent polymorphisms.

### Histopathology and Scanning electron microscopic - Energy dispersive spectrometer examination

Wound debridement and skin punch biopsy were performed simultaneously under appropriate illumination. Skin samples were fixed in 10% neutral buffered formalin, dehydrated in ethanol, embedded in paraffin and cut into 5 µm sections. After deparaffinization, the sections were evaluated for abnormalities of the skin and appendages by routine hematoxylin and eosin (H&E) staining. Epidermal innervation was further detected using immunohistochemistry staining with neuron-specific enolase (NSE) antibodies, a nerve fiber marker (1∶100; Santa Cruz Biotechnology, CA, USA).

We also obtained a spontaneously shed deciduous molar from the proband. This sample was examined was examined under various conditions by x-ray and Micro computed tomography (CT) (Inveon Micro-CT, Siemens, Germany) to detect abnormalities in structure and mineralization. After caries removal, half of the tooth was fixed with 4% paraformaldehyde, decalcified with 10% EDTA (pH 8.0), embedded in paraffin and routinely sectioned. Sections were deparaffinized and stained with H&E and Masson's stains. The other half of the tooth was used to prepare specimens for scanning electron microscopic (SEM) examination (FE-SEM, S-4800, Hitachi, Japan) and energy dispersive spectrometer (EDS) analysis (Pv72-45030LC, EDAX/AMETEK, USA; Igor Pro 6.0 software, Wavemetrics, USA).

### Genetic analysis

Blood samples were collected from all members of the family and control subjects. Genomic DNA was extracted from the blood samples using the QIAamp DNA Blood Mini Kit (Qiagen, USA) according to the manufacturer's protocol. Seventeen exons of the *NTRK1* gene were amplified using polymerase chain reaction (PCR) as previously reported with the Primer 3 on-line application (http://frodo.wi.mit.edu/primer3/input.htm) [10]. Primer sequences and optimal annealing temperature for each primer pair are available on request. The amplified fragments of *NTRK1* from individuals of the affected family and controls were gel-purified with a MinElute Gel Extraction Kit (Qiagen, USA. Sequencing analyses were performed with an ABI BigDyeTM terminator cycle sequencing ready reaction kit with AmpliTaq DNA polymerase on an ABI PRISMTM 377XLDNA sequencer (Applied Biosystems, USA). Finally, we analyzed the mutation in the NTRK1 at the genomic and protein levels using MegAlign 5.01 software (DNASTAR, USA). Protein modeling was conducted based on the recent data of NTRK1 structure in the Protein Data Bank (PDB ID code 4F0I, http://www.pdb.org.), and mutation-related residues were positioned in the three-dimensional structural model using the PyMOL Molecular Graphics System (Schrödinger L. version 1.3r1).
